# Genetic Diagnostic Elucidation of a Patient With Multiorgan Granulomas, Facial Peculiarities, and Psychomotor Retardation

**DOI:** 10.3389/fgene.2018.00355

**Published:** 2018-09-27

**Authors:** Daniel Soukup, Alma Kuechler, Joachim Roesler, Leopold Pichlmaier, Maximillian Eckerland, Margarete Olivier, Florian Stehling

**Affiliations:** ^1^Klinik für Kinderheilkunde I, Universitätsklinikum Essen, Essen, Germany; ^2^Institut für Humangenetik, Universitätsklinikum Essen, Essen, Germany; ^3^Klinik und Poliklinik für Kinder- und Jugendmedizin, Universitätsklinikum Carl Gustav Carus, Dresden, Germany; ^4^Klinik für Kinderheilkunde III, Universitätsklinikum Essen, Essen, Germany

**Keywords:** chronic granulomatous disease, lip, swelling, Williams–Beuren syndrome, microdeletion, compound heterozygosity, NCF-1, 7q11.23

## Abstract

We report the case of a 19-years-old patient who presented with a perplexing variety of symptoms which included remarkable facial features, intellectual disability, granulomatous upper lip swelling (previously diagnosed as Melkersson–Rosenthal syndrome), Crohn’s-like disease, non-productive cough, and a granulomatous mass localized in the left lung. Chronic granulomatous disease (CGD) was diagnosed using dihydrorhodamine 123 assay that showed low levels of phagocytic NADPH-oxidase. DNA sequencing revealed a heterozygous mutation in the *NCF-1* gene on chromosome 7. As remarkable facial features and psychomotor retardation are not associated with CGD, a more detailed genetic work-up using fluorescence *in situ* hybridization was performed. A microdeletion in 7q11.23 on one allele indicated Williams–Beuren syndrome (WBS). The *NCF-1* gene and its two pseudogenes are part of a highly repetitive region within 7q11.23 and are prone to recombination events and deletions. Such deletions can involve both the WBS critical region and the *NCF-1* wildtype gene, as was the case for our patient. The second allele of the *NCF-1* gene was affected by the frequent c.75.76delGT mutation that stems from a recombination of the *NCF-1* wildtype gene with one of its pseudogenes. In conclusion, patients with *NCF-1*-deficient CGD may also harbor microdeletions that result in WBS or other hereditary disorders; therefore, it is important to perform a thorough genetic analysis in order to initiate appropriate therapy for these patients.

## Background

Chronic granulomatous disease is a hereditary disorder that disrupts neutrophil activity. The disease is characterized by recurrent infections and a myriad of inflammatory complications (Holland, 2010). CGD is caused by mutations in genes responsible for the superoxide-generating phagocyte NADPH oxidase. This results in the absence of, or very low levels of enzyme activity in neutrophils, increasing susceptibility to infection and allowing for the proliferation of systemic granulomatous disease. The incidence of CGD is rare with 1 in 200 000 live births (Heyworth et al., 2003). More than two-thirds of all cases are linked to defects in the CYBB gene acquired in an X-linked recessive manner. The remaining cases are autosomal recessive inherited diseases caused by defects in *CYBA*, *NCF-1*, and *NCF-2* genes (Heyworth et al., 2003).

Williams–Beuren syndrome is a segmental aneusomy syndrome which results from a heterozygous deletion of numerous genes within the 7q11.23 region (Bayés et al., 2003). The phenotype of this syndrome includes growth retardation, facial dysmorphies, heart abnormalities, hyperacusis, infantile hypercalcemia, and abnormal gait. Cardiac abnormalities typically involve SVAS and peripheral pulmonary stenosis (Tassabehji, 2003). Patients have severe deficits in cognitive domains; personality is characterized by readiness to interact with strangers and overt friendliness. Approximately 70% exhibit attention deficit disorder (Tassabehji, 2003).

## Case Presentation

**FIGURE 1:**
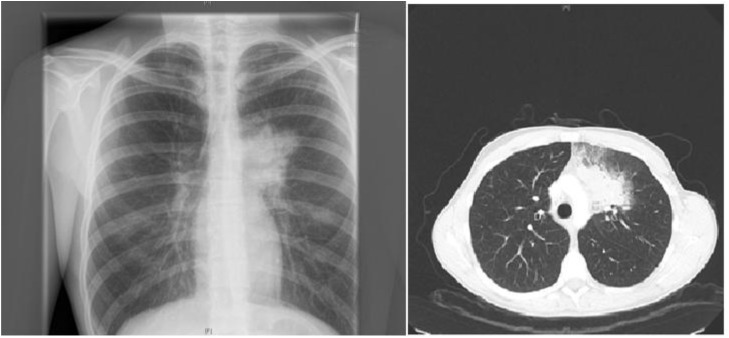
Patient’s X-ray (left) and chest-CT (right) showing a left upper lobe mass near the mediastinum resulting in a dry, non-productive cough.

**FIGURE 2:**
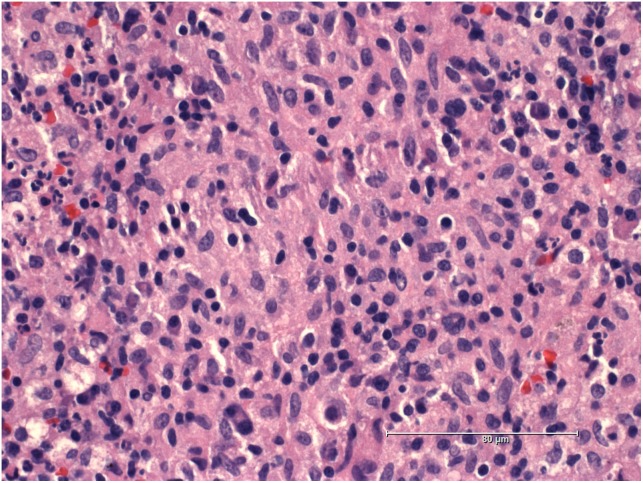
Histology sample of the intrathoracic mass showing granulomatous inflammation.

A 19-years-old patient was transferred to our pediatric hospital for further diagnostic analysis from a clinic specialized in adult lung diseases. The afebrile patient suffered from a dry, non-productive cough. An intrathoracic inflammatory granulomatous mass was localized by radiogram and computer tomography (**Figure 1**). Serum levels of soluble interleukin 2 receptor, neopterin, and angiotensin converting enzyme were negative. Bronchoalveolar lavage fluid showed lymphocytosis. Transbronchial biopsy revealed chronic, partly granulomatous inflammation with no signs of necrosis (**Figure 2**). No mycobacteria were found and the results were not typical for sarcoidosis. Steroid and antibiotic (cotrimoxazol) therapy resulted in rapid resolution of the intrathoracic mass.

**FIGURE 3:**
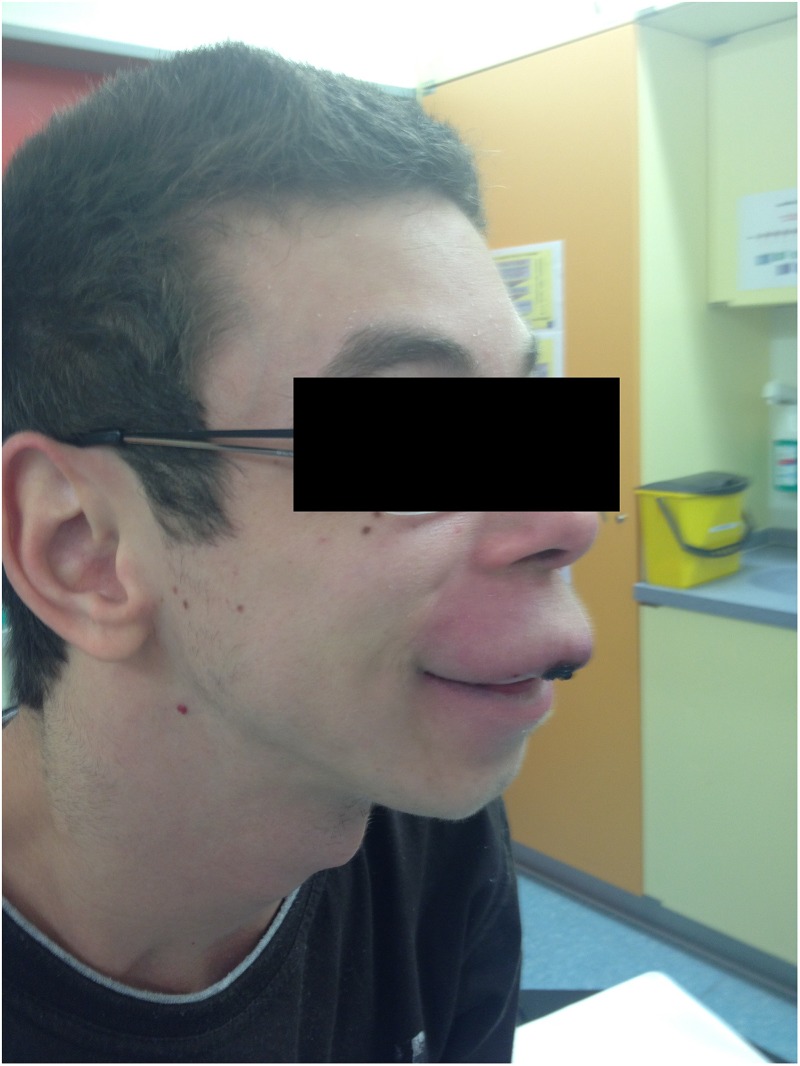
Facial phenotype of patient with upper lip granuloma and dysmorphic facies (symptoms of Williams–Beuren syndrome).

**FIGURE 4:**
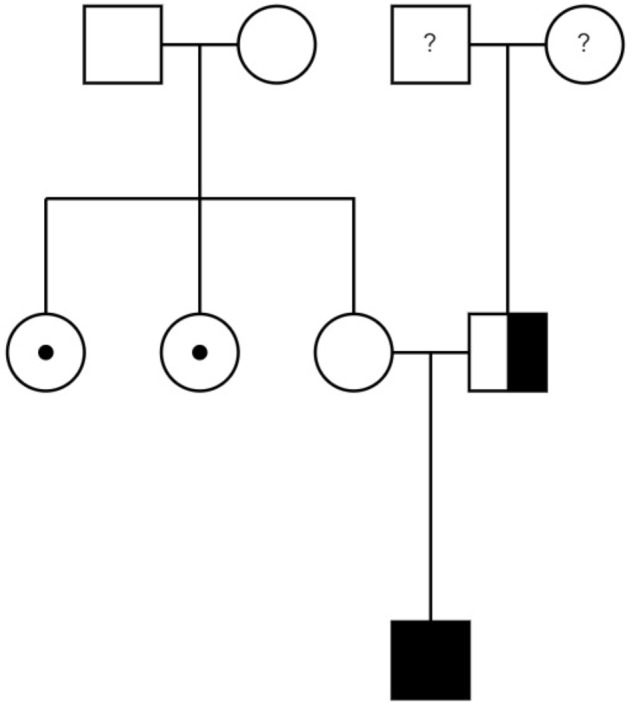
Family pedigree: the patient (only child) is at the bottom. Paternal information is missing – father does not reside with the family. The *NCF-1* gene was also heterozygous for the father. The mother’s sisters – marked with black dot – were both diagnosed with Crohn’s disease.

The patient’s past medical history included the diagnosis of a learning disability (patient currently attends special-needs school). Phenotypical dysmorphic facies had been noted including broad forehead, bi-temporal narrowing, strabismus, long philtrum, wide mouth, and large ear lobes (**Figure 3**). No further assessment of the patient’s cognitive abilities had been performed; however, clinically a mild intellectual disability was observed. MRS (OMIM 155900) and Crohn’s disease (OMIM 266600) had previously been diagnosed. MRS was histologically diagnosed at the age of 10 when the patient presented with swelling of the upper lip and granulomatous inflammation. However, typical features, such as lingua plicata or facialis paresis were not noted (Ang and Jones, 2002). Intense therapy with dapson, infliximab, azathioprine, and steroids led only to intermittent improvement. Crohn’s disease was diagnosed by screening at the age of 17, as two aunts had previously been diagnosed with Crohn’s disease (**Figure 4**). The patient had neither diarrhea nor significant gastrointestinal complaints. Colonoscopic biopsies revealed epithelial cell granulomas compatible with Crohn’s disease (see **Table 1**).

**Table 1:** Differential diagnosis of CGD and fundamental differences to other diagnoses.

Differential Diagnosis of CGD					
**Diagnosis**	**Site of symptoms**	**Recurrent infections**	**Pathogen spectrum**	**Impaired neutrophil respiratory burst**	**Other findings in contrast to CGD**
Chronic granulomatous Disease	Systemic	Yes	Opportune	Yes	–
Cystic fibrosis	Lung (infections)	Yes	Opportune	No	Infection limited to lung, bronchiectasis
Hyperimmunoglobulin E syndrome	Lung (infections)	Yes	Staphylococci	No	Characteristic facies, elevated IgE
G6PD deficiency	Systemic	Yes	Bacterial	Yes	Hemolytic anemia
Glutathione synthetase deficiency	Systemic	Yes	Bacterial	Yes	Hemolytic anemia, acidosis, ment. retard.
Crohn’s disease	Bowel	No	–	No	CGD has no extraintestinal symptoms

A multisystem granulomatous disease was suspected; therefore, the patient was tested for CGD using a dihydrorhodamine 123 (DHR) assay. This assay revealed a considerable decrease in NADPH-oxidase activity. Subsequent Sanger sequencing identified the most common mutation within the *NCF-1* gene (OMIM 608512: c.75.76delGT; p.Tyr26HisfsX26, chromosome 7, 7q11.23). The mutation was confirmed as heterozygous for the father, but surprisingly not for the mother. Unfortunately, symptomatic data for the father are unknown, as he does not reside with the family (**Figure 4**). The patient was administered the recommended prophylactic therapy of cotrimoxazol and itraconazol.

Nevertheless, neither learning disability nor facial dysmorphism are associated with CGD. The dysmorphism raised suspicion of WBS despite the absence of cardiac symptoms. Upon further genetic analysis, fluorescence *in situ* hybridization revealed microdeletions within 7q11.23 [46,XY.ish del(7)(q11.23q11.23)(ELN-)](OMIM 194050) with locus specific deletions; this confirmed the diagnosis of WBS.

## Discussion

The patient presented with granuloma formation in three different sites: lung, colon, and upper lip. This led to the differential diagnosis of CGD confirmed by DHR assay and *NCF-1* gene analysis. The clinical course of our patient differed from most CGD patients, due to the fact that *NCF-1*- (p47phox-) deficient CGD is generally associated with residual NADPH activity. This may explain why our patient had no history of life-threatening infection even while under immunosuppressive therapy aimed at treating previously suspected MRS. However, although *NCF-1*-deficient CGD generally follows a milder course than CGD lacking residual activity, some infectious manifestations can be equally as severe and life threatening as those contracted during the course of classical CGD, e.g., invasive aspergillus infections of the lungs (Heyworth et al., 2003).

The diagnosis of CGD alone did not account for all of our patient’s symptoms; instead, some underlying features such as facial dysmorphism and cognitive disability pointed toward WBS. We therefore expanded the genetic analysis and were able to confirm WBS. Our patient could then be treated appropriately for this disorder; treatment includes physical, developmental, and speech therapy (Morris, 1999; Bayés et al., 2003; Tassabehji, 2003). WBS usually occurs sporadically and is the result of heterozygous deletions of contiguous genes located close to the gene locus of CGD at 7q11.23. In the majority of patients, approximately 30 genes are missing (Bayés et al., 2003; Heyworth et al., 2003).

**FIGURE 5:**
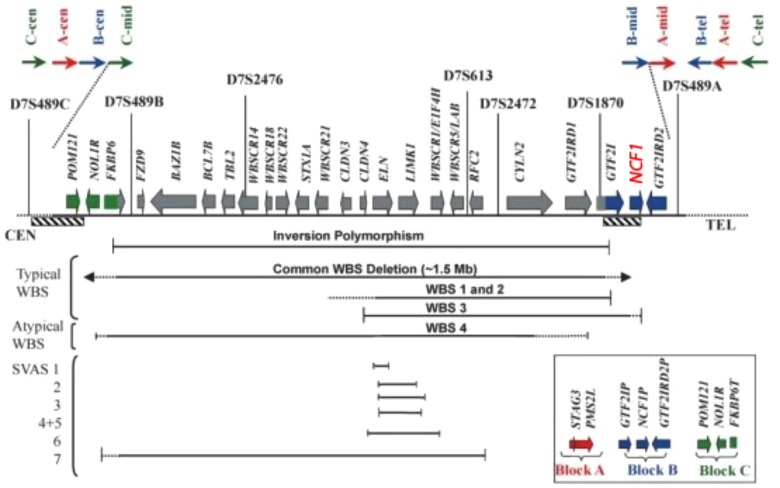
Transcript map of the Williams–Beuren syndrome critical region with the NCF1 gen (red) (Tassabehji, 2003).

The region depicted in **Figure 5** is highly repetitive already in non-human primates – compared to other mammals – and even more so in humans. Three large region-specific segmental duplications (centromeric, medial, and telomeric LCRs) with genetic variations have been identified. Each segmental duplication is composed of three differentiated blocks designated as “A,” “B,” and “C” (Bayés et al., 2003; Heyworth et al., 2003). Several genes and their unprocessed highly similar pseudogenes such as *NCF-1* are located centromeric and telomeric to the critical WBS region (see Merling et al., 2016) Depending on gene arrangement, several disorders may arise due to similar mechanisms. For example, a loop can be formed by pairing a region centromeric to the WBS stretch with a highly similar telomeric region. If the loop is removed enzymatically in a cellular DNA repair attempt, the microdeletion syndrome WBS results (Roesler et al., 2000). *NCF-1* can be involved in a loop larger than 1.5 Mb and also be deleted. Another mechanism with a similar result would be a pairing of such regions centromeric and telomeric to WBS between sister chromosomes 7 and subsequent unequal crossover. On a smaller scale, a recombination of the wildtype *NCF-1* gene with one of its pseudogenes could lead to a deletion or a partial deletion of the wildtype *NCF-1* gene (Roesler et al., 2000). Alternatively, after pairing with one of its pseudogenes, both harboring the GT-deletion at the beginning of exon 2, the wildtype gene could be converted into a pseudogene by DNA repair mechanisms in such a way that the GT-deletion moves into the wildtype gene. These possible rearrangements are difficult to detect by simple DNA sequencing of the critical *NCF-1* region, as the GT-deletions of the highly similar pseudogenes misrepresent the sequence information. The recombination of the wildtype with a pseudogene also generates a high number of *NCF-1*-deficient CGD carriers in the general population (ca. 1:700 in all ethnic groups) (Roesler et al., 2000).

These considerations apply to our patient in the following way: One allele of his 7q11.23 region harbors a large deletion of the critical WBS region that involves the *NCF-1* gene. The other allele harbors the frequent GT-deletion in exon 2 (c.75.76delGT) of the *NCF-1* wildtype gene. The mutation in *NCF-1* occurs then in the heterozygous state. Note that the WBS is normally caused by a *de novo* microdeletion, whereas the *NCF-1*-deficient CGD is inherited in an autosomal recessive manner. We find it remarkable that our patient is afflicted by two separate disorders that share a common background: Gene duplication (and triplication) with genetic variation in the region 7q11.23 during evolution and more recently as a result of recombination events between these regions.

It has been reported that a functional *NCF-1* gene increases the risk of hypertension in patients with WBS. It has also been found that the hemizygosity in *NCF-1* gene decreases this risk (Del Campo et al., 2006); yet, most of these patients do not exhibit CGD. Only four individuals with this condition have been reported. All were previously diagnosed with WBS, two presented with symptoms of chronic granulomas during adolescence when diagnosed with CGD, and one exhibited CGD symptoms during early childhood (Gilbert-Barness et al., 2009; Stasia et al., 2013).

## Conclusion

It is important to be aware of possible clinical indications suggestive of WBS in patients with *NCF-1*-deficient CGD. The identification of disease causing mutations using in-depth genetic analysis will facilitate the initiation of appropriate therapy for patients who are afflicted by these and other hereditary disorders.

## Ethics Statement

Written informed consent was obtained from the patient for publication of this case report and accompanying images. A copy of the written consent is available for review by the editor of this journal. The ethic committee approval has been obtained by institutional ethic committee of the medical faculty of the University Duisburg-Essen under file number 18-8226-BO and a copy of the approval is available for review by the editor of this journal.

## Author Contributions

DS made substantial contributions to conception and design and involved in drafting the manuscript. AK and JR made substantial contributions to the acquisition, analysis, and interpretation of the data. LP, ME, and MO involved in critically revising the manuscript for important intellectual content. FS made substantial contributions to conception and design, acquisition, analysis, and interpretation of the data, involved in revising the manuscript critically for important intellectual content, and gave the final approval of the version to be published.

## Conflict of Interest Statement

The authors declare that the research was conducted in the absence of any commercial or financial relationships that could be construed as a potential conflict of interest.

## Abbreviations

CGD: chronic granulomatous disease
CT: computed tomography
e.g.: exempli gratia
IFN: interferon
LCR: locus control region
MRS: Melkersson–Rosenthal syndrome
NADPH: reduced nicotinamide adenine dinucleotide phosphatase
NCF: neutrophil cytosolic factor
SVAS: supravalvular aortic stenosis
WBS: Williams–Beuren syndrome
